# Epitope Mapping of Antibodies Suggests the Novel Membrane Topology of B-Cell Receptor Associated Protein 31 on the Cell Surface of Embryonic Stem Cells: The Novel Membrane Topology of BAP31

**DOI:** 10.1371/journal.pone.0130670

**Published:** 2015-06-23

**Authors:** Won-Tae Kim, Hong Seo Choi, Hyo Jeong Hwang, Han-Sung Jung, Chun Jeih Ryu

**Affiliations:** 1 Institute of Anticancer Medicine Development, Department of Integrative Bioscience and Biotechnology, Sejong University, Seoul, Republic of Korea; 2 Division in Anatomy and Developmental Biology, Department of Oral Biology, Oral Science Research Center, BK21 PLUS Project, Yonsei University College of Dentistry, Seoul, Korea; 3 Oral Biosciences, Faculty of Dentistry, The University of Hong Kong, Hong Kong, Hong Kong SAR; Thomas Jefferson University, UNITED STATES

## Abstract

When located in the endoplasmic reticulum (ER) membrane, B-cell receptor associated protein 31 (BAP31) is involved in the export of secreted proteins from the ER to the plasma membrane. In a previous study, we generated two monoclonal antibodies (mAbs), 297-D4 and 144-A8, that bound to surface molecules on human embryonic stem cells (hESCs), but not to surface molecules on mouse embryonic stem cells (mESCs). Subsequent studies revealed that the mAbs recognized BAP31 on the surface of hESCs. To investigate the membrane topology of BAP31 on the cell surface, we first examined the epitope specificity of 297-D4 and 144-A8, as well as a polyclonal anti-BAP31 antibody (α-BAP31). We generated a series of GST-fused BAP31 mutant proteins in which BAP31 was serially deleted at the C- terminus. GST-fused BAP31 mutant proteins were then screened to identify the epitopes targeted by the antibodies. Both 297-D4 and 144-A8 recognized C-terminal residues 208–217, while α-BAP31 recognized C-terminal residues 165–246, of BAP31 on hESCs, suggesting that the C-terminal domain of BAP31 is exposed on the cell surface. The polyclonal antibody α-BAP31 bound to mESCs, which confirmed that the C-terminal domain of BAP31 is also exposed on the surface of these cells. Our results show for the first time the novel membrane topology of cell surface-expressed BAP31 as the extracellular exposure of the BAP31 C-terminal domain was not predicted from previous studies.

## Introduction

B-cell receptor-associated protein 31 (BAP31) is known to be a 28 kDa integral endoplasmic reticulum (ER) membrane protein that is expressed ubiquitously [1–3]. Composed of three membrane-spanning segments and a 13 kDa cytoplasmic tail containing an extended coiled-coil region [4], BAP31 promotes the vesicular transport of transmembrane proteins, such as class I major histocompatibility complex, immunoglobulin D, cellubrevin, teteraspanins, cytochrome P450, and CD11b/CD18 [5–11]. Thus, BAP31 is a chaperone/quality control factor that participates in the transport and quality control of membrane proteins. Many studies have also shown that the C-terminus of BAP31 is exposed on the cytoplasmic side of the ER and cleaved by caspase-8 in response to apoptosis-inducing stimuli [3, 4, 6, 8, 12–16]. The mitochondrial protein Fis1 and BAP31 complex that spans the mitochondria–ER interface serves as a platform to activate the initiator procaspase-8 [14, 15]. During apoptosis, the exposed C-terminus of BAP31 are targeted by caspases, and the cleavage product, p20BAP31, which remains in the ER membrane, transmits the apoptosis signal [17]. Thus, BAP31 is also an important regulator of apoptosis on the ER membrane.

We previously generated monoclonal antibodies (mAbs) against surface molecules of human embryonic stem cells (hESCs) using a modified decoy immunization strategy [18]. Among the mAbs produced, 297-D4 recognized BAP31 on the surface of hESCs and some cancer cell lines, including A375 (human malignant melanoma), SH-SY5Y (human neuroblastoma), Colo-205 (human colon carcinoma), and HepG2 (human hepatocellular carcinoma) [19]. Subsequent studies revealed that BAP31 positively regulates hESC adhesion, stemness, and survival by interacting with epithelial cell adhesion molecule (EpCAM) on the surface [19]. To investigate the membrane topology of BAP31 on the cell surface, we first examined the epitope specificity of 297-D4 for BAP31. Epitope mapping of 297-D4 and two other anti-BAP31 antibodies suggests that the C-terminal domain of cell surface-expressed BAP31 is exposed on the extracellular side. The result is unexpected because the cytoplasmic side of ER membrane proteins is generally preserved even after translocation to the plasma membrane [20]. To our knowledge, this is the first report showing the unexpected membrane topology of cell surface-expressed BAP31.

## Materials and Methods

### Cell Cultures

H9 hESC line (WiCell, Madison, WI, USA) was cultured on irradiated mouse embryonic fibroblast (MEF) feeder cells as described previously [18, 19, 21]. Briefly, hESCs were maintained in DMEM/F12 medium supplemented with 20% Knockout serum replacement (Invitrogen, Seoul, Korea), 0.1 mM 2-mercaptoethanol, 1% non-essential amino acid, 1 mM glutamine, 100 U/ml penicillin G, 100 μg/ml streptomycin, and 4 ng/ml basic fibroblast growth factor (PeproTech, Rocky Hill, NJ, USA). hESCs were subcultured every 5 days with 1 mg/ml collagenase IV (Invitrogen, Seoul, Korea). Mouse embryonic stem cell (R1) line was cultured and maintained as described previously [19, 21].

### Preparation and induction of GST-fusion protein

Serially truncated BAP31 proteins were expressed as fusion proteins with GST proteins. The coding sequences of serially truncated and whole BAP31 genes were synthesized by polymerase chain reaction from the BAP31-myc-KKEE plasmid using 5’-primer and various 3’-primers and subcloned into the EcoRI/SalI sites of pGEX4T-2 (GE Healthcare, Seoul, Korea) to yield the expression plasmids [22]. All primer sequences are listed in **Table 1**. Each expression plasmid was confirmed by DNA sequencing, and introduced into *E*. *coli* DH5α cells to express the GST-BAP31 fusion proteins. The expression of the fusion proteins was induced by 0.1 mM isoprophyl-β-D-thiogalactopyranoside at 32°C for 6 h. The induced bacterial cells were washed with pre-chilled phosphate-buffered saline (PBS, pH 7.4), incubated with acetone on ice for 5 min, and lysed in 1% SDS supplemented with 100 μg/ml phenylmethanesulfonyl fluoride (PMSF) for 2 min at room temperature (RT). Proteins were clarified by centrifugation, and their concentration was measured by bicinchoninic assay (Thermo Scientific, Rockport, IL, USA). The cell lysates were subjected to 12.5% SDS-PAGE, stained with Coomassie brilliant blue R-250, and analyzed by western blot analysis [23].

**Table 1:** Primer sequence for the generation of BAP31 deletion mutants.

N-terminus		CCT GAA TTC CCA TGA GTC TGC AGT GGA CT
C-terminus	124	CGT GTC GAC TTA CGA AAT GAG AGT CAC
	164	CAA GTC GAC TTA GTC AAC AGC AGC TCC
	207	GGC GTC GAC TTA GTT TTC AGC TTT CTC
	217	CTT GTC GAC TTA CTC AGA CTG CTT
	227	TGC GTC GAC TTA CAG CAA GCG GTC
	237	CAT GTC GAC TTA TAC TGC AGC CTG
	245	AGG GTC GAC TTA TTC CTT CTT GTC
	246	AGG GTC GAC TTA CTC TTC CTT CTT GTC

### Flow Cytometry

Flow cytometric analysis was performed using antibodies as described previously [18, 19, 21]. Briefly, H9 hESCs were incubated with appropriate primary antibodies for 30 min at 4°C. The primary antibodies used were anti-TRA-1-60, anti-TRA-1-81, α-BAP31 (Santa Cruz Biotechnology sc-48766, Santa Cruz, CA, USA), goat anti-BAP31 (Santa Cruz Biotechnology sc-18579), mouse anti-BAP31 (Santa Cruz Biotechnology sc-365347), 297-D4, or 144-A8 antibodies. The cells were then further incubated with fluorescein isothiocyanate (FITC)-conjugated anti-mouse IgG, anti-rabbit IgG, anti-goat IgG, or anti-mouse IgM (BD Biosciences) in PBA (1% bovine serum albumin, 0.02% NaN3 in PBS), depending on the isotype of the primary antibodies. Propidium iodide (PI, 25 μg/ml) was used to detect and eliminate dead cells from the samples. Negative cells for PI staining were gated to exclude the dead cells and analyzed on a Becton-Dickinson FACSCalibur.

### Antibody-antibody competition binding assay

H9 hESCs were pre-incubated with 5 μg/ml of isotype control antibody, α-BAP31, or biotin-labeled 297-D4 at 4°C for 30 min. The cells were then incubated with 2 μg/ml of competing antibodies 297-D4 and 144-A8 for α-BAP31 binding, or 2 μg/ml of 144-A8 for biotin-labeled 297-D4 binding at 4°C for 30 min. Antibody binding was measured by flow cytometric analysis. The secondary antibodies used were Alexa 488-conjugated rabbit IgG or phycoerythrin-conjugated streptavidin. The percent of binding inhibition was calculated from mean fluorescence intensity. The percent inhibition of competing antibodies was normalized against each antibody binding in the presence of isotype control antibody. The graph presents the mean values of at least three independent determinations ± standard deviation. Statistical analysis used the Student’s t-test.

### Cell surface biotinylation, immunoprecipitation, and western blotting

Cell surface biotinylation, immunoprecipitation, and western blotting were performed as described previously [18, 19, 21]. Briefly, biotin-labeled H9 hESCs or R1 mESCs were treated with lysis buffer (25 mM Tris-HCl, pH 7.5, 250 mM NaCl, 5 mM EDTA, 1% Nonidet P-40, 2 μg/ml aprotinin, 100 μg/ml PMSF, 5 μg/ml leupeptin, and 1mM NaF) at 4°C for 30 min, and nuclei were removed by centrifugation. To immunoprecipitate the antigens recognized by the antibodies, the lysates were pre-cleared with Protein G plus-Sepharose (Santa Cruz Biotechnology, Santa Cruz, CA, USA), incubated with 297-D4, 144-A8 or α-BAP31 at 4°C overnight, and further incubated with Protein G plus-Sepharose. The bound proteins were eluted from the beads by heating at 100°C for 10 min after extensive washing with lysis buffer. Eluted proteins were fractionated by SDS-PAGE and then transferred to a nitrocellulose membrane for western blotting. The membrane was blocked in 5% skim milk in PBST (PBS containing 0.1% Tween 20) at RT for 1 h and incubated with SA-HRP (GE Healthcare, Seoul, Korea) at RT for 1 h. The biotinylated proteins were visualized using the ECL detection reagent (GE Healthcare, Seoul. Korea) after extensive washing. For western blot analysis, proteins on polyacrylamide gels were transferred to a nitrocellulose membrane and incubated with 297-D4, 144-A8, or α-BAP31 antibodies followed by HRP-conjugated anti-mouse IgG or anti-rabbit IgG for 1 h at RT as described before [19]. The membranes used were stripped at 50°C for 30 min in stripping buffer (62.5 mM Tris-Cl, pH6.7, 100 mM 2-mecaptoethanol, 2% SDS). The membranes were then washed and subjected to western blotting again. The immunoblots were visualized as described above.

## Results

### Two mAbs 297-D4 and 144-A8 recognize cell surface-expressed BAP31

**Fig 1:**
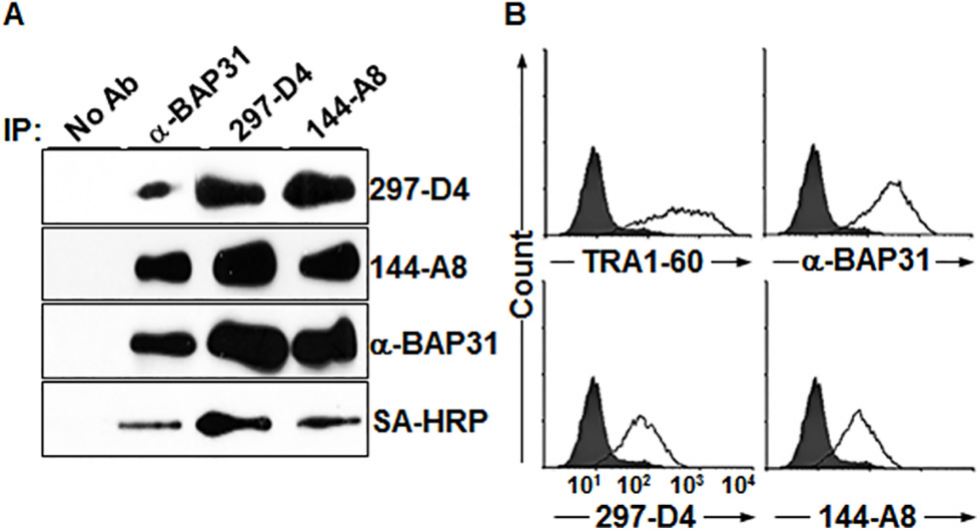
MAb 297-D4 and MAb 144-A8 recognize BAP31 on the cell surface of hESCs. (A) Immunoprecipitation of biotinylated hESCs with α-BAP31, 297-D4, and 144-A8 antibodies. Immunoprecipitates were detected by western blots with the indicated antibodies or SA-HRP. (B) Flow cytometric analysis of hESCs with anti-TRA-1-60, α-BAP31, 297-D4, or 144-A8 antibodies.

In a previous study, we generated a panel of mAbs against the surface molecules of hESCs and found that the mAb 297-D4 (IgG1, κ) recognizes BAP31 on the surface of hESCs [18, 19], suggesting that BAP31, an ER resident protein, is also expressed on the cell surface. To confirm that BAP31 is expressed on the cell surface of hESCs, the cell surface molecules of hESCs were biotinylated and subjected to immunoprecipitation and western blotting with 297-D4 and α-BAP31, a commercially available rabbit polyclonal antibody against BAP31. Another mAb 144-A8 (IgG1, κ), an independently isolated mAb against a surface molecule of hESCs, was also included in this analysis. Proteins immunoprecipitated with 297-D4, 144-A8, or α-BAP31 were readily detected by western blot analysis with 297-D4, 144-A8, or α-BAP31 (**Fig 1A,** the first, second, and third panels), indicating that both 297-D4 and 144-A8 recognize the BAP31 protein. The immunoprecipitated BAP31 proteins were also detected by horseradish peroxidase-conjugated streptavidin (SA-HRP) (**Fig 1A,** the fourth panel), indicating that the BAP31 proteins recognized by the three antibodies are expressed on the cell surface. Flow cytometric analysis with propidium iodide (PI)-negative hESCs also revealed that the BAP31 proteins recognized by the two mAbs and α-BAP31 are expressed on the surface of hESCs (**Fig 1B**). Thus, the results indicate that the two mAbs, 297-D4 and 144-A8, and α-BAP31 recognize cell surface-expressed BAP31.

### Fine epitope mapping of 297-D4, 144-A8, and α-BAP31

**Fig 2:**
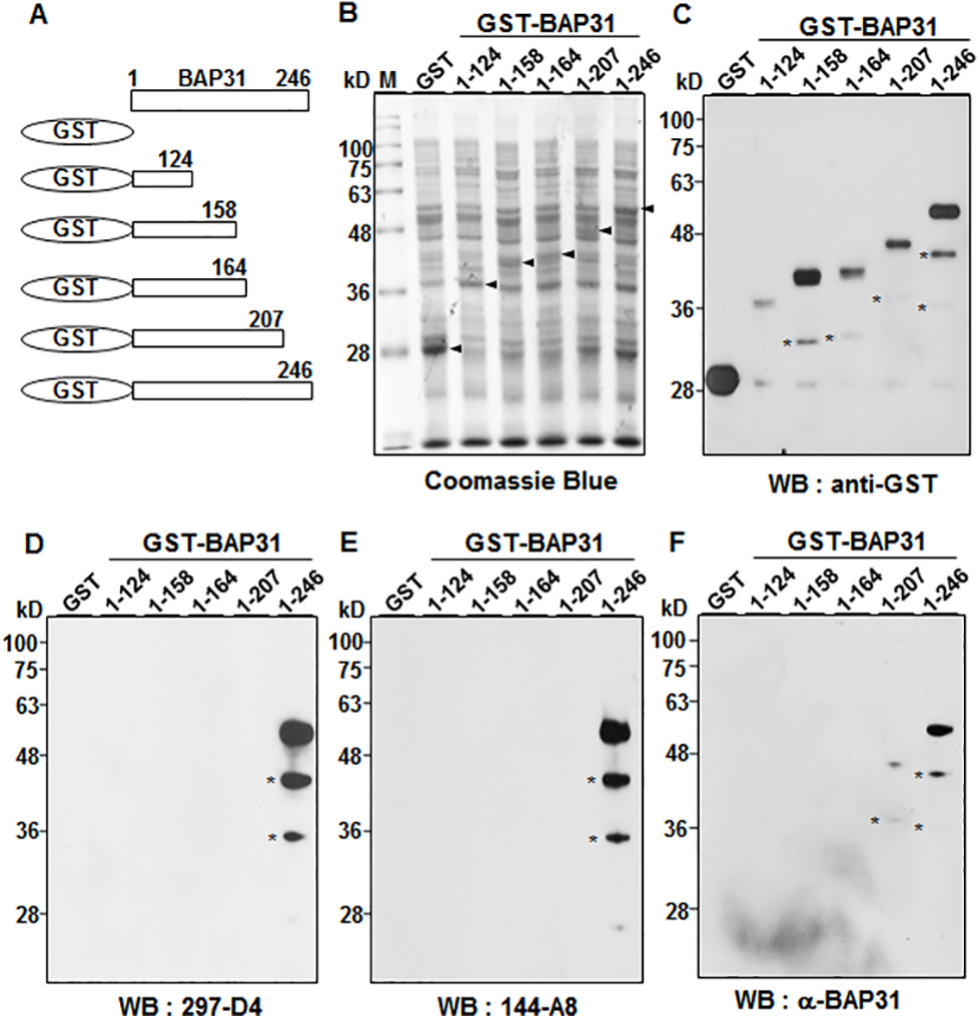
Epitope mapping of 297-D4, 144-A8, and α-BAP31 antibodies. (A) Schematic diagram of recombinant BAP31 fragments (residues 1–124, 1–158, 1–164, 1–207, and 1–246) used in this study. (B) Individual fusion proteins were expressed in bacteria as fusion proteins with GST tag at the N-terminus and stained with Coomassie brilliant blue after SDS-PAGE. (C-F) Western blot analysis of GST-BAP31 fusion proteins with anti-GST (C), 297-D4 (D), 144-A8 (E), and α-BAP31 (F) antibodies. The asterisks indicate partial degradation products of GST-BAP31 fusion proteins.

Previous studies have shown that BAP31 has three transmembrane domains within its amino terminus, and the short hydrophilic amino terminus of BAP31 is exposed in the ER lumen while the 13 kDa C-terminal domain of BAP31 is exposed on the cytoplasmic side [3, 4, 6, 8, 12–16]. Therefore, we expected that cell surface-expressed BAP31 would have the same membrane topology, i.e., with a cytoplasmic C-terminal domain. However, cell surface-expressed BAP31 was recognized in immunoprecipitation and flow cytometric analysis by α-BAP31, which was raised against residues (157–246) mapping within the C-terminal cytoplasmic domain of BAP31 (**Fig 1A and 1B**). The results imply that the C-terminal domain of BAP31 is not located in the cytoplasm, but is exposed on the cell surface when the protein localizes on the plasma membrane. 297-D4 and 144-A8 also recognized cell surface-expressed BAP31 (**Fig 1B**), suggesting that the epitopes of both mAbs are also located on the C-terminal domain of BAP31. To determine the exact epitopes of 297-D4, 144-A8, and α-BAP31, a series of C-terminal deletion mutants of BAP31 gene were synthesized by PCR and fused to glutathione S-transferase (GST) gene to construct a series of expression plasmids (**Fig 2A**). Each plasmid was introduced into *E*. *coli*, and the protein extracts of the recombinant cells were analyzed by SDS-PAGE. The expression of serial deletion mutants of BAP31 were visualized by Coomassie Blue staining and analyzed by western blot analysis with an anti-GST antibody. All serial deletion mutants of GST-BAP31 fusion proteins were readily expressed and detected (**Fig 2B and 2C**). The partially degraded forms of the serial deletion mutants of GST-BAP31 fusion proteins were also detected below the main GST-BAP31 fusion proteins (**Fig 2C**). The same recombinant *E*.*coli* lysates were then subjected to western blot analysis with 297-D4, which recognized the full-length form (residues 1–246), but not the deletion mutant forms (residues 1–207, 1–164, 1–158, and 1–124) (**Fig 2D**). The same result was also obtained with 144-A8 (**Fig 2E**). The results suggest that 297-D4 and 144-A8 recognize the linear epitopes located between residues 208–246 of BAP31. The polyclonal antibody α-BAP31 recognized the full length and the deletion mutant with residues 1–207, but not the deletion mutants (residues 1–124, 1–158, and 1–164) (**Fig 2F**), suggesting that the epitopes of α-BAP31 are within the residues 165–246 of BAP31.

**Fig 3:**
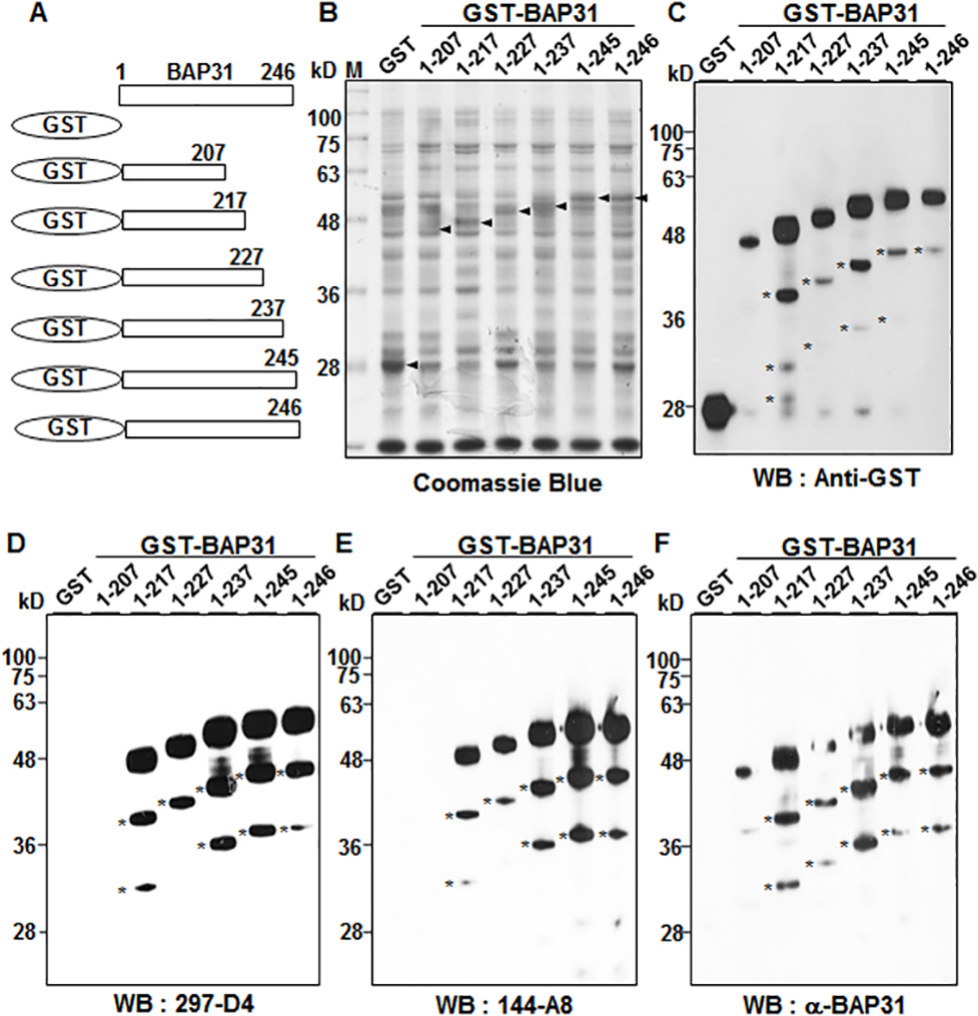
Fine epitope mapping of 297-D4, 144-A8, and α-BAP31 antibodies. (A) Schematic diagram of recombinant BAP31 fragments (residues 1–207, 1–217, 1–227, 1–237, 1–245, and 1–246) used in this study. (B) Individual fusion proteins were expressed in bacteria as fusion proteins with GST tag at the N-terminus and stained with Coomassie brilliant blue after SDS-PAGE. (C-F) Western blot analysis of GST-BAP31 fusion proteins with anti-GST (C), 297-D4 (D), 144-A8 (E), and α-BAP31 (F) antibodies. The asterisks indicate partial degradation products of GST-BAP31 fusion proteins.

To find the fine epitopes of 297-D4, 144-A8, and α-BAP31, residues 207–246 of BAP31 were further dissected and expressed as GST-BAP31 fusion proteins. The expression of the BAP31 deletion mutants (residues 1–207, 1–217, 1–227, 1–237, 1–245, and 1–246) of GST-BAP31 proteins were confirmed by western blot analysis with an anti-GST antibody (**Fig 3A, 3B and 3C**). The partially degraded forms of the serial deletion mutants of GST-BAP31 fusion proteins were again detected below the main GST-BAP31 fusion proteins (**Fig 3C**). 297-D4 recognized the serial deletion mutants (residues 1–217, 1–227, 1–237, 1–245 and 1–246), except for residues 1–207 (**Fig 3D**). The same result was also obtained with 144-A8 (**Fig 3E**). The results indicate that 297-D4 and 144-A8 recognize the linear epitopes between residues 208–217 of BAP31. The polyclonal antibody α-BAP31 recognized all deletion mutants (residues 1–207, 1–217, 1–227, 1–237, 1–245 and 1–246), although it did not recognize the deletion mutants (residues 1–124, 1–158 and 1–164) (**Figs 2F and 3F**), confirming that the epitopes of α-BAP31 are scattered throughout the residues 165–246 of BAP31.

### 297-D4 and 144-A8 compete with each other

**Fig 4:**
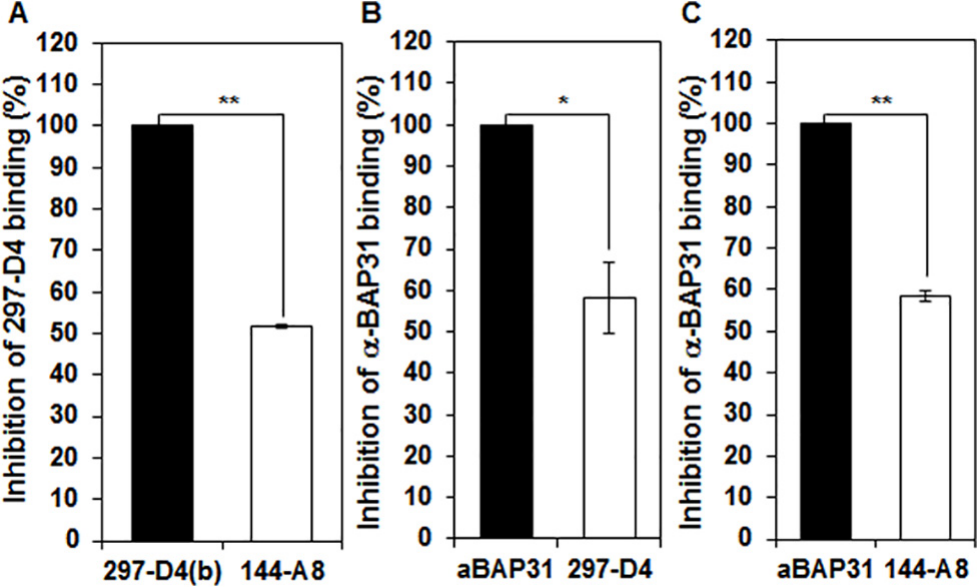
Antibody-antibody competition binding assay. (A) hESCs were preincubated with biotinylated 297-D4 in the presence of isotype control antibody and 144-A8 prior to the addition of phycoerythrin-conjugated streptavidin. (B,C) hESCs were preincubated with α-BAP31 in the presence of isotype control antibody, 297-D4 (B), 144-A8 (C) prior to the addition of FITC-conjugated anti-rabbit IgG. Antibody binding to hESCs was then analyzed by flow cytometry. The percent of binding inhibition was calculated from mean fluorescence intensity. Statistical analysis used the Student’s t-test (*p<0.05; **p<0.001).

Both 297-D4 and 144-A8 recognized the residues 208–217 of BAP31 in the epitope mapping analysis. To further confirm whether the two mAbs recognize the overlapping epitope on the native BAP31 protein, we performed antibody competition binding assay by using flow cytometric analysis. hESCs were preincubated with biotinylated 297-D4 before the addition of competing antibody 144-A8. 297-D4 binding was inhibited by approximately 49% with 144-A8 (**Fig 4A**), indicating that the two mAbs have overlapping epitopes on the native BAP31 protein. When hESCs were preincubated with α-BAP31 before the addition of competing antibodies 297-D4 or 144-A8, α-BAP31 binding was also inhibited by approximately 42% with both antibodies (**Fig 4B and 4C**). The result suggests that the predominant epitope recognized by the polyclonal α-BAP31 overlapped with the epitopes of 297-D4 and 144-A8.

### Mouse Bap31 is also expressed on the surface of mESCs

**Fig 5:**
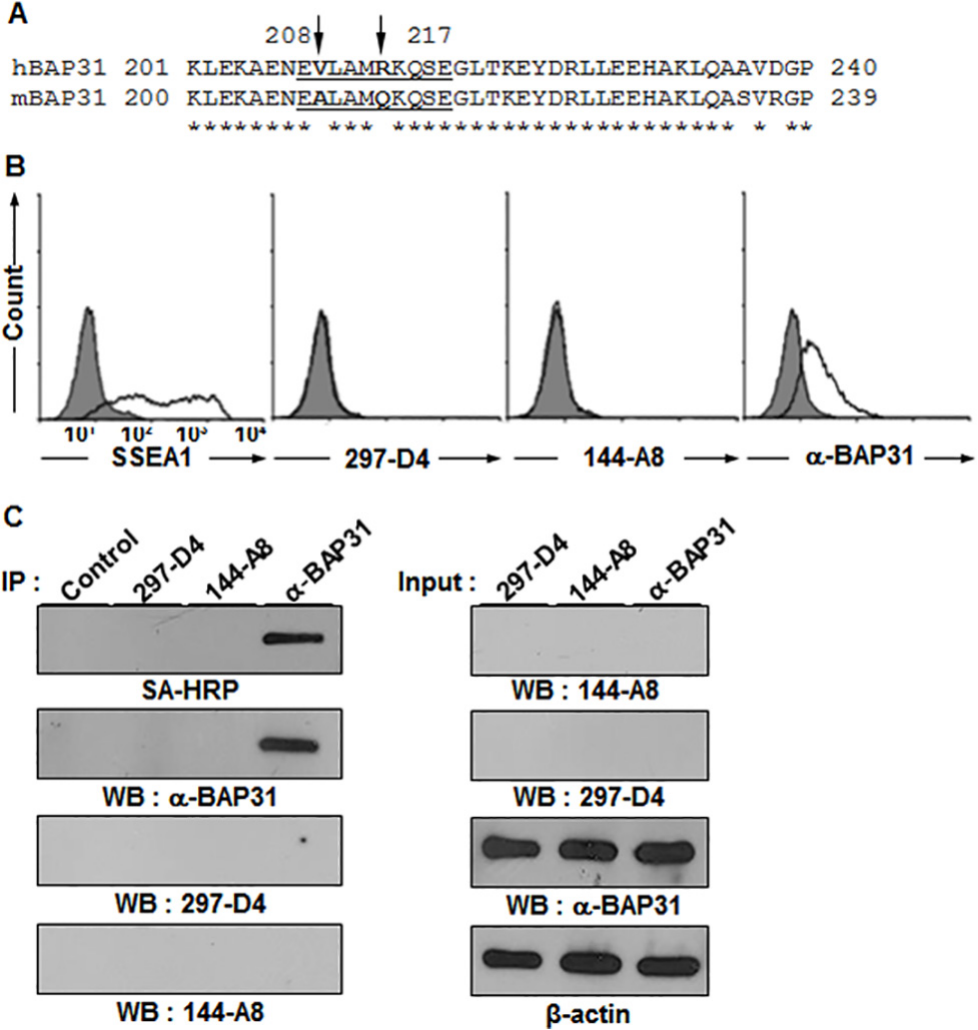
BAP31 is also expressed on the surface of mESCs. (A) The epitope sequences of 297-D4 and 144-A8 are different between hESCs and mESCs. The partial C-terminal amino acid sequences were aligned between human and mouse BAP31. The epitope sequences are underlined, and the different amino acids are indicated by arrows. (B) Flow cytometry analysis of mESCs with anti-SSEA-1, 297-D4, 144-A8, and α-BAP31 antibodies. (C) Immunoprecipitation of mESCs with 297-D4, 144-A8, and α-BAP31 antibodies. Cell surface proteins of mESCs were biotinylated, immuoprecipitated, and analyzed by SA-HRP or western blot with α-BAP31 (left panels). The input samples were also analyzed by western blots (right panels). β-actin was used as a loading control.

We found by using 297-D4 that BAP31 is expressed on the surface of hESCs, but not on the surface of mESCs [19], suggesting that mouse Bap31 is not expressed on the surface of mESCs. However, sequence alignment showed that the residues at 209 and 213, within the epitope of 297-D4, were different between human BAP31 and mouse Bap31 (**Fig 5A**). Therefore, we postulate that Bap31 is expressed on the surface of mESCs, as well, but it is not detected due to the sequence difference between human and mouse BAP31. Although the epitope of the polyclonal α-BAP31 overlapped with the epitope of 297-D4, its binding to hESCs was not completely inhibited by 297-D4. This suggests that some epitopes recognized by the polyclonal α-BAP31 are outside of the epitope of 297-D4. As shown in **Fig 1A and 1B**, cell surface-expressed BAP31 on hESCs was recognized by α-BAP31. Therefore, we examined the surface expression of Bap31 on mESCs by using the polyclonal α-BAP31 antibody. As expected, α-BAP31 was able to bind to mESCs but 297-D4 was not (**Fig 5B**). To further confirm whether Bap31 is also expressed on the surface of mESCs, cell surface proteins of mESCs were biotinylated and subjected to immunoprecipitation and western blotting with α-BAP31, 297-D4, or 144-A8. α-BAP31 alone was able to immnunoprecipitate Bap31 from mESCs (**Fig 5C** left, the second, third, and fourth panels). SA-HRP staining further confirmed that α-BAP31 alone was able to recognize and immunoprecipitate mouse Bap31 on the cell surface (**Fig 5C** left, the first panel). Thus, the results confirm that BAP31 is expressed on the surface of both hESCs and mESCs.

### Proposed model for cell surface-expressed BAP31

**Fig 6:**
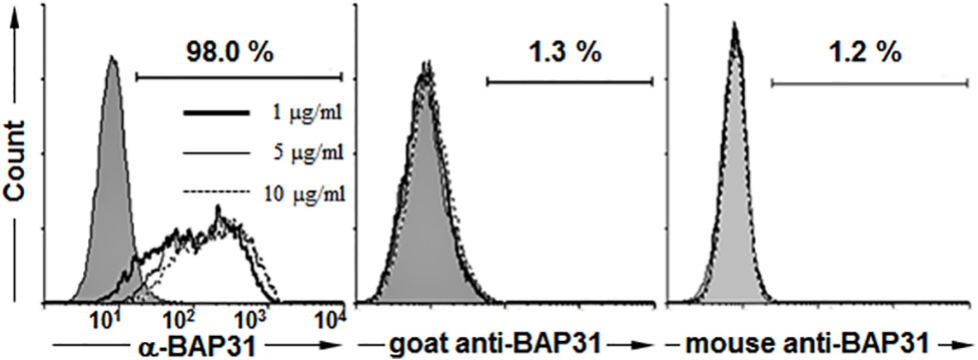
Flow cytometric analysis of the percent expression of cell surface-expressed BAP31 on H9 hESCs. The cell surface expression of BAP31 was examined by flow cytometric analysis with various concentrations (1, 5, or 10 μg/ml) of rabbit (residues 165–246), goat (residues 125–158), or mouse (residues 137–161) anti-BAP31 antibodies. Shown are the percentages of BAP31-positive cells at the concentration of 10 μg/ml of antibodies.

BAP31 contains three transmembrane domains within its amino terminus that provide topology in the ER membrane; the short amino terminus of BAP31 is exposed in the ER lumen, and the 13 kDa C-terminal domain of BAP31 is exposed on the cytoplasmic side [3, 8, 13, 15]. The same membrane topology of BAP31 was expected in the plasma membrane because the cytoplasmic side of ER membrane proteins is usually preserved after translocation to the plasma membrane [20]. Our results indicate, however, that the C-terminal domain of BAP31 is not located in the cytoplasm but is exposed on the cell surface. To further confirm whether the C-terminal domain of BAP31 is exposed on the cell surface, we analyzed the cell surface expression of BAP31 on H9 hESCs by flow cytometric analysis with various concentrations of α-BAP31, a goat polyclonal anti-BAP31 antibody (specific for the internal region), or a mouse monoclonal anti-BAP31 antibody (specific for residues 137–161). Epitope analysis showed that the goat polyclonal anti-BAP31 antibody recognized residues 125–158 in the western blot analysis (S1 Fig). The cell surface expression of BAP31 was detected in 98% of the total population with α-BAP31, while it was hardly detected with the goat and mouse antibodies (Fig 6). The results indicate that the C-terminal domain of BAP31 from residues 165–246 is exposed and recognized by α-BAP31 on the cell surface, while the C-terminal domain from residues 125–161 is not recognized by the goat and mouse antibodies.

**Fig 7:**
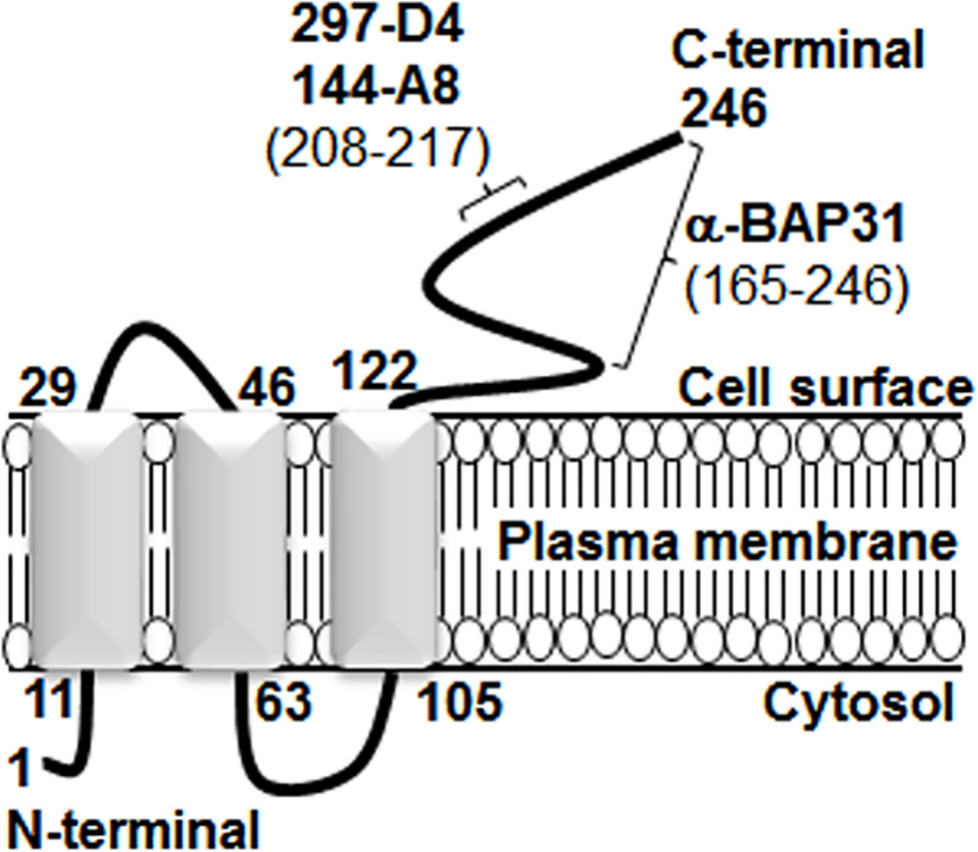
Proposed model for the membrane topology of BAP31 on the cell surface of hESCs and mESCs.

Next, we predict and propose a new membrane topology for cell surface-expressed BAP31, based on the present study and server-based computing program (www.predictprotein.org) (**Fig 7 and Table 2**). The new topology suggests that the cytosolic regions of BAP31 are amino acid residues 1–10 and 64–104, and the extracellular regions are amino acid residues 30–45 and 123–246. According to the amino acids’ characteristics (e.g., hydrophobicity, hydrophilicity) the computing program detected three membrane helices and two loops for the best model. Although the sidedness of BAP31 C-terminal domain is different between the ER membrane and the plasma membrane, the structure of the three membrane helices and the two loops is consistent with the previous finding [3]. On the other hand, the present antibody studies also suggest the possibility that the C-terminal domain from residues 125–161 is not available to antibodies on the cell surface, although the domain is predicted to be on the extracellular side of the plasma membrane.

**Table 2:** Prediction of transmembrane helices and topology of cell surface-expressed BAP31.

Positions	Segments
1–10	Inside region 1
11–29	Transmembrane domain 1
30–45	Outside region 1
46–63	Transmembrane domain 2
64–104	Inside region 2
105–122	Transmembrane domain 3
123–246	Outside region 2

## Discussion

We previously found that BAP31 is expressed on the surface of hESCs, [19] despite the fact that the protein is known as a chaperone and quality control factor mainly located in the ER membrane [1–3, 5, 11, 19, 24, 25]. BAP31 contains a KKEE ER retention signal at its C terminus, although some studies showed that mutation of the KKEE sequence does not alter the ER distribution of BAP31 [1, 3, 11]. We also attempted to find any alterations in BAP31 sequences that are involved in the translocation of BAP31 into the plasma membrane of hESCs. We cloned and sequenced 19 BAP31 cDNAs from H9 hESCs and NT-2/D1, and found that most clones had normal standard BAP31 sequences (data not shown). Although we found some sporadic point mutations in some clones, they were not consistent among the different clones, which suggest that the mutations are minor variants or artifacts. Thus, we could not find any major variant forms of BAP31 that could be responsible for the translocation to the plasma membrane of hESCs.

We found that cell surface translocation of BAP31 was not limited to hESCs. By flow cytometry, we could detect BAP31 on the surface of some cancer cell lines, such as A375, SH-SY5Y, Colo-205, and HepG2 [19]. A low level of BAP31 was also detected on the surface of U87-MG, NCI-H69, and A549 cells [19]. Previous studies also showed that a small portion of BAP31 is located at the plasma membrane of human colonic epithelial cells and human foreskin fibroblast [8, 26]. Therefore, it is conceivable that BAP31 protein may be initially inserted into the ER membrane, but a small portion of BAP31 protein may be translocated into the plasma membrane, depending on the cell type.

In this study, we found that two mAbs, 297-D4 and 144-A8, recognized cell surface-expressed BAP31 (**Fig 1**), and their epitopes are located between residues 208–217 of BAP31 (**Figs 2D, 2E, 3D and 3E**). The results suggest that the C-terminal domain of cell surface-expressed BAP31 is exposed on the extracellular side. We also found that cell surface-expressed BAP31 was recognized by the commercial polyclonal α-BAP31 antibody. Information from the supplier indicates that α-BAP31 was raised against residues 157–246 of BAP31, and the present study further defined that the epitope for this polyclonal antibody was scattered throughout amino acid residues 165–246, mapping within the C-terminal cytoplasmic domain of BAP31 (**Figs 2F and 3F**). Taken together, the results suggest that the C-terminal domain (residues 165–246) of cell surface-expressed BAP31 is exposed on the extracellular side. On the other hand, cell surface-expressed BAP31 was not recognized by the goat and mouse anti-BAP31 antibodies specific for residues 125–161 (**Fig 6**), although the epitope regions of the antibodies are also predicted to be on the extracellular side (**Table 2**). It is possible to speculate that the region is not available to the antibodies because it is associated with neighboring molecules on the cell surface. Another possibility is that the epitopes of the antibodies are simply inaccessible in the native conformation of BAP31. A proposed model for cell surface-expressed BAP31 is presented (**Fig 7**).

Secretory and membrane proteins enter the membrane trafficking system by translocation from the cytoplasm into the ER, and proteins are packaged into carrier vesicles that deliver proteins to the Golgi complex and sorted into secretory vesicles, which transport proteins to the plasma membrane [27]. The cytoplasmic region of membrane proteins remains on the cytoplasmic side during the process of cell surface translocation [20, 27]. Unexpectedly, the present study suggests another membrane topology for BAP31, which is different from the previous finding (**Fig 7**) [3]. The phenomenon can be explained by the multiple topologies of BAP31. Many studies have shown that multiple or dynamic topologies are found in some membrane proteins, such as cystic fibrosis transmembrane conductance regulator, ductin, aquaporin-1, and P-glycoprotein [28–34]. Even though the functions of multiple topological proteins are not clearly identified [28, 34], different topologies may be associated with different functions. Our previous finding showed that cell surface-expressed BAP31 colocalizes with EpCAM [19], suggesting that the function of cell surface-expressed BAP31 may be associated with EpCAM. The exact role of cell surface-expressed BAP31 remains to be determined.

## Supporting Information

S1 Fig Epitope mapping of the goat polyclonal anti-BAP31 antibody. (A) Schematic diagram of recombinant BAP31 fragments (residues 1–124, 1–158, 1–164, 1–207, and 1–246) used in this study. (B,C) Individual fusion proteins were expressed in bacteria as fusion proteins with GST tag at the N-terminus and transferred to nitrocellulose membranes after SDS-PAGE. GST fusion proteins were analyzed by western blots with anti-GST antibody (B) and the goat polyclonal anti-BAP31 antibody (C), specific for the internal region of BAP31. The asterisks indicate partial degradation products of GST-BAP31 fusion proteins.
(TIF)
