# Correction: Epitope Mapping of Antibodies Suggests the Novel Membrane Topology of B-Cell Receptor Associated Protein 31 on the Cell Surface of Embryonic Stem Cells: The Novel Membrane Topology of BAP31

**DOI:** 10.1371/journal.pone.0170145

**Published:** 2017-01-06

**Authors:** Won-Tae Kim, Hong Seo Choi, Hyo Jeong Hwang, Han-Sung Jung, Chun Jeih Ryu

In Fig 5A, the N-terminal residue of the underlined epitope is incorrectly listed as Glutamate (E) instead of Glutamine (Q). Please see the corrected Fig 5 here.

**Fig 5:**
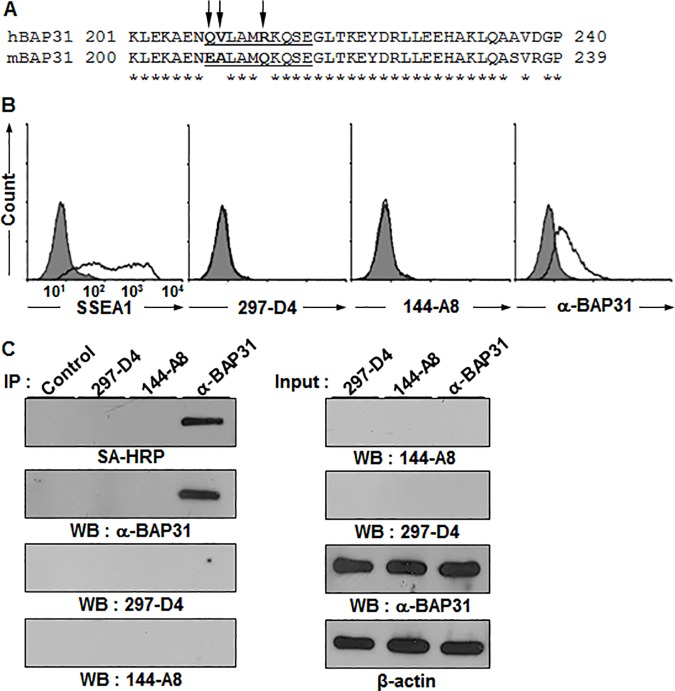
BAP31 is also expressed on the surface of mESCs. (A) The epitope sequences of 297-D4 and 144-A8 are different between hESCs and mESCs. The partial C-terminal amino acid sequences were aligned between human and mouse BAP31. The epitope sequences are underlined, and the different amino acids are indicated by arrows. (B) Flow cytometry analysis of mESCs with anti-SSEA-1, 297-D4, 144-A8, and α-BAP31 antibodies. (C) Immunoprecipitation of mESCs with 297-D4, 144-A8, and α-BAP31 antibodies. Cell surface proteins of mESCs were biotinylated, immuoprecipitated, and analyzed by SA-HRP or western blot with α-BAP31 (left panels). The input samples were also analyzed by western blots (right panels). β-actin was used as a loading control.
