# Supplementary figures and images for: Epitope Mapping of Antibodies Suggests the Novel Membrane Topology of B-Cell Receptor Associated Protein 31 on the Cell Surface of Embryonic Stem Cells: The Novel Membrane Topology of BAP31
Source: PLoS One. 2015 Jun 23;10(6):e0130670. doi: 10.1371/journal.pone.0130670 (PMC4478030; doi:10.1371/journal.pone.0130670)

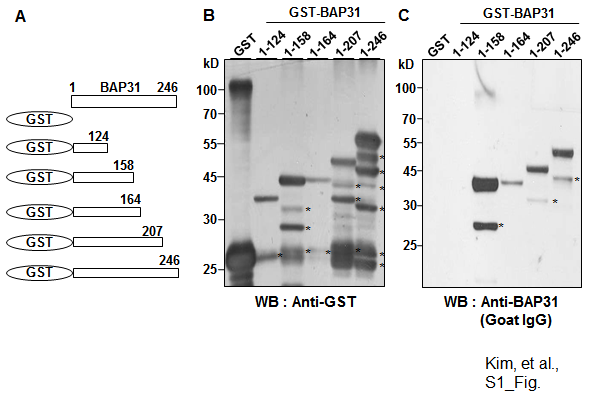

Supplement: S1 Fig — (A) Schematic diagram of recombinant BAP31 fragments (residues 1–124, 1–158, 1–164, 1–207, and 1–246) used in this study. (B,C) Individual fusion proteins were expressed in bacteria as fusion proteins with GST tag at the N-terminus and transferred to nitrocellulose membranes after SDS-PAGE. GST fusion proteins were analyzed by western blots with anti-GST antibody (B) and the goat polyclonal anti-BAP31 antibody (C), specific for the internal region of BAP31. The asterisks indicate partial degradation products of GST-BAP31 fusion proteins. (TIF) [file pone.0130670.s001.tif]
